# Insights into the genetic aetiology of diabetes in Africa

**DOI:** 10.1016/j.ebiom.2026.106265

**Published:** 2026-04-27

**Authors:** Alisha N. Wade, Carolyn Padoa, Adebowale Adeyemo, Inês Barroso

**Affiliations:** a Research in Metabolism and Endocrinology, Department of Internal Medicine, School of Clinical Medicine, Faculty of Health Sciences, University of the Witwatersrand, Johannesburg, South Africa; b MRC/Wits Rural Public Health and Health Transitions Research Unit, School of Public Health, University of the Witwatersrand, Johannesburg, South Africa; c Division of Endocrinology, Diabetes and Metabolism, Perelman School of Medicine, University of Pennsylvania, Philadelphia, PA, USA; d Department of Chemical Pathology, School of Pathology, Faculty of Health Sciences, University of the Witwatersrand, Johannesburg, South Africa; e South Africa and National Health Laboratory Services, Johannesburg, South Africa; f Center for Research on Genomics and Global Health, National Human Genome Research Institute, National Institutes of Health, Bethesda, MD, USA; g Exeter Centre of Excellence for Diabetes Research (EXCEED), University of Exeter Medical School, Exeter, UK

**Keywords:** Diabetes, Genetics, Glycated haemoglobin, Genetic score, Variant interpretation, Africa

## Abstract

Despite the high public health burden of diabetes in Africa, research into its genetic aetiology has been slow, limiting the continent's ability to benefit from an emerging era of diabetes precision medicine. Some progress is evident. In monogenic diabetes, where a molecular diagnosis enables tailored treatment, two cases from Africa successfully illustrate this approach despite the absence of routine affordable genetic testing, and control data from diverse African populations. Although limited, genome-wide association studies from African populations have discovered novel African-specific type 2 diabetes risk variants. Additionally, application of a type 1 diabetes genetic score helped define a novel type of insulin deficient non-autoimmune diabetes in sub-Saharan Africa. Multiple challenges remain, including interpretation of glycated haemoglobin, a frequently used diabetes biomarker, which is impacted by genetic variants common in the African continent. We review these issues, outline barriers to implementing diabetes precision medicine, and highlight areas for future development.

## Introduction

In Africa, the prevalence of all forms of diabetes is increasing at an alarming rate, with the age-standardised prevalence in adults expected to change from 5% in 2024 to 5.9% in 2050- an increase from 24.6 million adults to approximately 60 million.^1^ Although the region has the lowest age-adjusted diabetes prevalence globally, the expected 142% increase between 2024 and 2050 is the largest worldwide.^1^ While the incidence of type 1 diabetes (T1D) in those less than 15 years in the region is the lowest in the world at an estimated 2 per 100,000, the 605,000 people of all ages estimated to have died prematurely from T1D in Africa is the third highest globally, decreasing prevalence and potentially underestimating disease burden.^2^ Despite the impact of diabetes in the region, the paucity of high-quality genetic studies in African countries means few individuals can benefit from the advances in molecular diagnosis of diabetes that reduce misclassification and facilitate optimal therapeutic approaches.^3^ This review summarises current knowledge on the genetics of diabetes in African populations and highlights the need for continental African genetic research to better define molecular risk factors, develop population-specific risk models and improve diagnostic and therapeutic approaches.

This is the fourth in a Series of four papers about diabetes in sub-Saharan Africa (papers 1-3 appear in *The Lancet Diabetes & Endocrinology*). All papers in the Series are available at https://www.thelancet.com/series-do/diabetes-in-sub-saharan-africa

## Molecular basis of diabetes in Africa

Diabetes is broadly classified into monogenic diabetes, T1D, type 2 diabetes (T2D), gestational diabetes (GDM) and other specific types of diabetes. Its classification is usually based on clinical characteristics such as age at onset, family history of diabetes, body mass index (BMI), insulin dependence and sensitivity, and laboratory features such as C-peptide levels and the presence of pancreatic β-cell autoantibodies. However, the overlap of clinical and laboratory features between different types of diabetes presents a significant diagnostic challenge and illustrates the importance of appropriate genetic testing to improve classification.

### Monogenic diabetes

The term “monogenic diabetes” refers to a group of conditions caused by penetrant pathogenic variants in individual genes in each affected family.^4^ The main types of monogenic diabetes are neonatal diabetes (NDM) and maturity onset diabetes of the young (MODY). NDM presents within the first 6 months of life and has a reported incidence range of 0.00021%–0.0048% of live births, depending on the population.^4^ MODY normally presents in lean individuals before the age of 25 years who have a strong family history of diabetes, and is reported to affect 1 in 10,000 adults and 1 in 23,000 children worldwide.^4^

Little is known about the epidemiology of monogenic diabetes across Africa. The reported incidence of NDM varies widely between populations, with higher incidence in countries with higher rates of consanguinity.^5^ One of the first studies of NDM in Africa reported an incidence of 4.8 per 100,000 live births in Sudan.^6^ This high incidence cannot be extrapolated to other African countries, as Sudan has a high degree of ethnic diversity, represents only a subset of genetic diversity across Africa and has one of the highest rates of consanguineous marriage.^7^ Globally, mutations in over 40 different genes have been shown to cause different forms of NDM and while mutations in *EIF2AK3 and KCNJ11* were first described in children from Morocco and East Africa respectively, little is known about the main causes of NDM in Africa.^4^^,^^6^^,^^8^^,^^9^ In the Sudanese study mentioned above, the most common causes of NDM (18.9%) were recessive mutations in *EIF2AK3* causing Wolcott–Rallison syndrome.^6^ This aligns with an earlier study which included families from 79 countries where the degree of parental consanguinity affected the relative frequency of NDM causative mutations.^10^ NDM was due to a mutation in *EIF2AK3* in 24% of children of consanguineous parents, whereas, mutations in *ABCC8* and *KCNJ11* accounted for only 12% of these cases. In contrast, in children of non-consanguineous parents, mutations in *ABCC8* and *KCNJ11* accounted for 46% of NDM cases.^10^ Consistent with this, mutations in *INS*, *ABCC8* and *KCNJ11* account for over 50% of NDM cases in Europe and North America.^4^

MODY prevalence in continental African populations is unknown. Monoallelic pathogenic mutations in 11 different genes have been reproducibly reported to cause MODY in patients from different countries.^11^ To date, there are no published data on the prevalence of each of these in African countries, although cases of GCK-MODY and HNF1A-MODY have been identified in Tunisia and Cameroon, respectively.^12^^,^^13^ In addition, mutations in *WFS1* leading to a syndromic form of diabetes with optical atrophy and hearing loss were observed in two Moroccan families.^14^ Although *WFS1* is not a MODY gene, mutations in the gene are a recognised cause of monogenic syndromic diabetes.

In summary, neither the prevalence nor genetic aetiology of monogenic diabetes are well characterised across different African countries, and the condition is likely to be underdiagnosed due to limited genetic testing.

### Type 1 diabetes

T1D is a heterogeneous disease arising from the complex interaction between genetic and environmental factors that triggers islet autoimmunity and the destruction of insulin secreting pancreatic β-cells. T1D commonly presents in childhood with a peak incidence at 5–9 years of age although, in sub-Saharan Africa, a second peak in onset has been observed after the age of 20 years.^15^ While latent autoimmune diabetes in adults (LADA—defined by insulin independence for ≥6 months, age at onset ≥30 years and diabetes-associated autoantibody positivity) is an important consideration in the context of adult-onset T1D, it only accounted for a small percentage (17.8%) of cases in the second peak. Removal of these cases from analyses had little effect on the outcomes suggesting a different aetiology of T1D for this group.^15^ A recent study including participants from South Africa, Cameroon and Uganda, confirmed that 65.1% of cases with young-onset (age <30 years), insulin-treated and clinically diagnosed T1D, have non-autoimmune insulin deficient diabetes.^16^ These results suggest that in sub-Saharan Africa T1D is a very heterogeneous condition including classical autoimmune T1D cases, as well as a novel type of non-autoimmune insulin deficient diabetes.^16^

Globally, the primary genetic susceptibility locus for T1D is the major histocompatibility complex with the HLA class II *DRB1* and *DQB1* genes conferring the strongest genetic susceptibility.^17^ Studies investigating HLA associations with T1D in African populations (Table 1) are limited and primarily originate from north African countries whose populations exhibit strong Middle Eastern and Mediterranean influences. These studies confirm previous susceptibility and protective allele findings from European-ancestry studies, although allele frequencies may differ. Of note, high resolution typing identified DR04:05 as a risk allele, with a frequency of 13.4–19.3%, in Ethiopian, Malian, Moroccan and Sudanese populations whereas the most common European risk allele is DR04:01 (approximately 28.1%).^17^^,^^20^^,^^22^^,^^23^^,^^25^ In addition, similar to Asian populations, DR9 is common (9.4–10.6%) and predisposes to T1D in sub-Saharan African populations while it is rare (0.2–0.8%) in European-ancestry populations.^17^^,^^22^^,^^24^ A novel association of T1D with DQB1∗02:02 was seen in an Egyptian and Malian population.^18^^,^^22^ Furthermore, DR10 was protective in a Sudanese and Ethiopian population whereas no association was seen in European-ancestry populations.^17^^,^^20^^,^^25^

**Table 1:** Association of HLA class II DRB1 and DQB1 alleles with T1D in African and European samples.

Population	Sample size	Risk alleles/haplotypes	Protective alleles/haplotypes	Genotyping	Reference
Egyptian	85 cases and 113 controls	DQB1∗0201 DQB1∗0202 DQB1∗0302	DQB1∗0601	PCR-SSOP	Mosaad et al., 2012^18^
Egyptian	68 cases and 120 controls	DR3-DQB1∗02 DR4-DQB1∗0302		PCR-SSOP	El-Amir et al., 2019^19^
Ethiopian	202 cases and 166 controls	DR3-DQB1∗02 DRB1∗0405-DQB1∗02 DRB1∗0405-DQB1∗0302 DRB1∗0401-DQB1∗0302	DRB1∗0404-DQB1∗04 DR15-DQB1∗0602 DR11/12/13-DQB1∗0301 DR13-DQB1∗0603 DR7-DQB1∗0303 DRB1∗0403-DQB1∗0302 DR1/10-DQB1∗0501 DR7-DQB1∗02 DR13-DQB1∗0604	PCR-SSOP	Gudeta et al., 2025^20^
Ethiopian (Amhara)	188 cases and 152 controls	DRB1∗03:01 DRB1∗04	DRB1∗15	PCR-SSP	Balcha et al., 2020^21^
Malian	99 cases and 200 controls	DRB1∗03:01 DRB1∗04:05 DRB1∗09:01 DQB1∗02:01 DQB1∗02:02 DQB1∗03:02	DRB1∗15:03 DQB1∗04:02 DQB1∗05:02 DQB1∗06:02 DQB1∗06:03	NGSgo-MX11-3 system	Noble et al., 2024^22^
Moroccan	90 cases and 139 family members as controls	DRB1∗03:01 DRB1∗04:05 DQB1∗02:01 DQB1∗03:02	DRB1∗11 DRB1∗15 DQB1∗06	PCR-SSP and PCR-SSOP	Drissi Bourhanbour et al., 2015^23^
South African (Zulu)	47 cases and 630 controls	DRB1∗0301 DRB1∗04 DRB1∗09 DQB1∗02 DQB1∗0302	DRB1∗0302	PCR-SSP	Pirie et al., 2001^24^
Sudanese	56 cases and 198 controls	DRB1∗03:01 DRB1∗04:02 DRB1∗04:05	DRB1∗10:01 DRB1∗13:02 DRB1∗15:02 DRB1∗15:03	Roche 454 GS Junior System and MiSeq instrument	Ibrahim et al., 2021^25^
Tunisian	137 cases and 258 controls	DRB1∗03 DRB1∗04	DRB1∗11 DRB1∗15	PCR-SSOP	Hajjej et al., 2019^26^
European ancestry	607 families consisting of two parents and at least two affected siblings	DRB1∗0301-DQA1∗0501-DQB1∗0201 DRB1∗0401-DQA1∗0301-DQB1∗0302 DRB1∗0404-DQA1∗0301-DQB1∗0302 DRB1∗0402-DQA1∗0301-DQB1∗0302 DRB1∗0405-DQA1∗0301-DQB1∗0302	DRB1∗1501-DQA1∗0102-DQB1∗0602 DRB1∗1104-DQA1∗0501-DQB1∗0301 DRB1∗0701-DQA1∗0201-DQB1∗0303 DRB1∗1303-DQA1∗0501-DQB1∗0301 DRB1∗1401-DQA1∗0101-DQB1∗0503	PCR-based SSOP system	Erlich et al., 2008^17^

The HLA class I genes (*HLA-A*, *HLA-B*, *HLA-C*) are poorly studied in relation to T1D in Africa (Table 2) making direct comparisons challenging due to differences in HLA typing methodologies, alleles investigated, HLA resolution, inconsistencies in nomenclature, and small sample sizes (68–99 cases and 120–200 control participants). Novel class I allele associations were seen in a Malian cohort, except for A∗24:02 which is a known risk allele globally.^22^

**Table 2:** Associations of HLA-A, HLA-B and HLA-C alleles with T1D in African and European studies.

Population	Sample size	Risk alleles	Protective alleles	Genotyping method	Reference
Egyptian	68 cases and 120 controls	B∗8		PCR-SSP	El-Amir et al., 2019^19^
Malian	99 cases and 200 controls	B∗27:05 A∗24:02 A∗29:02 C∗02:02	A∗30:01 A∗74:01 B∗42:01 B∗53:01	NGSgo-MX11-3 system	Noble et al., 2024^22^
European (Denmark; Human Biological Data Interchange; Joslin Diabetes Center; Sardinia) and four T1DGC networks (Asia Pacific; European; North American; United Kingdom)	1753 T1D probands and 1585 affected family-based control chromosomes	A∗24:02 A∗02:01 B∗18:01 C∗05:01	A∗11:01 A∗32:01 A∗66:01 B∗07:02 B∗44:03 B∗35:02 C∗16:01 C∗04:01	PCR-based SSOP system	Noble et al., 2010^27^

More than 75 non-HLA susceptibility genes have been identified in European-ancestry populations, predominantly by genome-wide association studies (GWAS).^28^ In Africa, genetic research has relied on candidate gene studies.^29^, ^30^, ^31^, ^32^, ^33^, ^34^ While associations of *IL-10*, *MALAT1, RAGE* and *VDR* with T1D have been seen in Egyptian and South African populations, respectively, due to their small sample size (ranging from a total sample size of 184–354) and more lenient p-value thresholds, false-positive associations cannot be ruled out.^29^^,^^31^^,^^33^^,^^34^ In addition, weaker genetic associations may be missed due to lack of power. Notably, a preliminary GWAS performed on an Ethiopian cohort with 236 cases and 200 controls found no genome-wide significant associations with T1D, emphasising the need for larger-scale studies across the African continent.^21^

In summary, although unique genetic associations with T1D have been seen in different African populations, progress in the field has been limited by the small sample sizes (ranging from a total sample size of 184–677) and lack of GWAS highlighting the need for larger powered studies across Africa to inform T1D risk determination, improved diagnostic accuracy and the possibility of tailored treatment plans.

### Type 2 diabetes

T2D is the most common type of diabetes, often accounting for 80–90% of all diabetes cases globally. Non-genetic factors (e.g. age, obesity and behaviour) and over 600 loci contribute to T2D risk in multi-ancestry studies.^35^ Progress in identifying genetic risk factors in Africa has been slow, with initial studies including linkage and candidate gene studies that evaluated GWAS-associated loci from predominantly European ancestry populations.^36^, ^37^, ^38^, ^39^

The first set of GWAS of T2D in Africa were published in 2019 (Table 3). The first GWAS included 5231 individuals enrolled from Nigeria, Ghana, and Kenya, in the Africa America Diabetes Mellitus Study (AADM) and identified two genome-wide significant loci- *TCF7L2* (a well-known T2D locus) and *ZRANB3*, a novel T2D locus.^40^ Functional data from zebrafish and *in vitro* cell line models supported a role for *ZRANB3* in T2D risk and aetiopathogenesis. The discovery of *ZRANB3* added to the growing list of T2D risk loci that were first discovered in under-studied populations, such as *KNCQ1* in East Asians and *SLC16A11* in Mexicans and other Latin Americans.^42^, ^43^, ^44^ The study also showed transferability of 32 established GWAS-associated T2D loci, thereby identifying a set of T2D loci that are shared with other populations.

**Table 3:** Significant genetic associations with T2D in African studies.

Population	Sample size	Gene	Variant (Effect allele)	Reference
Discovery: Ghanaians, Nigerians and Kenyans Replications: South African (Zulu)	Discovery: 5231 (2342 T2D cases, 2889 controls) Replication: 2578 (1602 T2D cases, 976 controls)	*TCF7L2* *ZRANB3* *HMGA2*	rs7903146 (T) rs1465146591 (A) rs138066904 (C)	Adeyemo et al.,^40^
Meta-analysis GWAS of South African Zulu, Ghanaians, Nigerians and Kenyans	4347 (2633 T2D cases, 1714 controls)	*TCF7L2* *AGMO*	rs7903146 (T) rs17746147 (C)^a^ rs73284431 (G)	Chen et al.,^41^

a Second independent variant at the *TCF7L2* locus.

The second GWAS was a meta-analysis that included 4347 participants from the AADM study and two South African studies and identified *TCF7L2* as the most significantly associated locus, thereby confirming its role in West Africans, East Africans, and Southern Africans.^41^ Notably, fine-mapping of the *TCF7L2* locus identified one signal shared between Europeans and Africans (indexed by rs7903146) and a distinct African-specific signal (indexed by rs17746147). The study also detected one novel candidate locus near *AGMO* (indexed by rs73284431) which is monomorphic in most non-African populations and distinct from previously reported signals in the region.

While these studies have been included in larger collaborative meta-analyses for T2D, no other GWAS for T2D have been published specifically from African populations. A few studies investigated glycaemic traits such as fasting glucose, fasting insulin, HbA1c or HOMA indices.^45^^,^^46^ In general, the findings of GWAS for such traits only have a partial overlap with GWAS for T2D though they can elucidate important pathophysiology. The more recent of these studies did a GWAS of fasting glucose, fasting insulin, insulin resistance (HOMA-IR) and beta cell function (HOMA-B), in ∼10,000 individuals from Burkina Faso, Ghana, Kenya and South Africa within the context of the Africa Wits-INDEPTH Partnership for Genomics Studies (AWI-Gen).^46^ The findings included genome-wide significant associations of fasting glucose with *ANKRD33B*, fasting insulin with *WDR7* and HOMA-IR with *ADMATS16* and *B4GLAT6*, the first two of which are novel associations. Several known GWAS-associated loci with fasting glucose were replicated, including variants in *GCK-YTK6*, *SLC2A2* and *THORLNC*.

In summary, there is a paucity of genome-wide association studies of T2D in Africa. However, the few studies that have been done yielded novel discoveries while confirming several previous findings of risk loci in other populations. The broad genetic diversity of African populations and the heterogeneity in environment, diet and behavioural factors suggest that more studies are needed to fully characterise the genetic risk factors of T2D in Africa.

### Gestational diabetes

GDM is defined as diabetes first diagnosed after 15 weeks of gestation that was not present prior to conception, and that is not another type of diabetes first detected during pregnancy.^47^ GDM affects approximately 14% of pregnancies in Africa, on par with the global estimates.^48^

Globally, genetic studies of GDM are lagging relative to T2D. Since GDM is thought to have shared genetic aetiology with T2D, early studies focused on testing established T2D risk loci (e.g. *CDKAL1, TCF7L2, KCNJ11, GCK, MTRN1B*) and found they are also associated with GDM.^49^ Few of these studies included Africans, with the only studies from the continent from Egypt and South Africa testing candidate SNPs for association with GDM.^50^^,^^51^ However, the sample sizes in these studies were modest (ranging from total sample size of 160–447) and there were therefore the inherent problems of lack of power and the risk of both false-positive and false-negative findings.

More recently, a genome-wide multi-ancestry meta-analysis, including 5485 women with GDM and 347,856 without GDM identified 5 loci associated with GDM (mapping to/near *MTNR1B, TCF7L2*, *CDKAL1*, *CDKN2A-CDKN2B* and *HKDC1*).^52^ However, the 1.7% African ancestry individuals included in the study were African Americans from the United States, so in addition to having European admixture, they do not represent the genetic diversity observed in continental African populations.

To date, the largest GDM GWAS (2332 cases and 131,109 women without GDM) from the Finnish FinnGen study, identified 13 loci associated with GDM.^53^ The authors suggested that the genetic aetiology of GDM should be partitioned into a T2D risk component, and a component primarily reflecting mechanisms disrupted by pregnancy.^53^ However, no African participants were included in these analyses and therefore the shared and distinct genetic aetiology of GDM and T2D in Africa remains unexplored.

## Impact of genetics on diabetes precision medicine

**Fig. 1:**
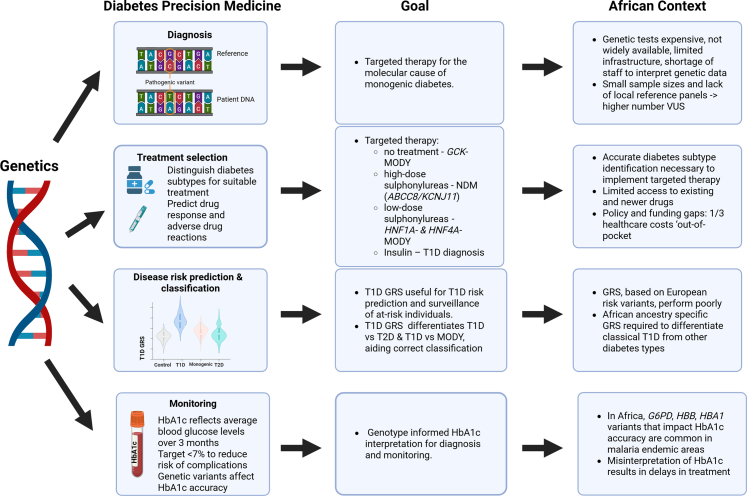
**Diabetes Precision Medicine: General Goals and African Context.** Four pillars of precision medicine are represented corresponding to diagnosis, treatment selection, disease risk prediction and classification and monitoring. For each pillar the broader global goal is described as well as the African context. Created in BioRender. Barroso, I. (2026) https://BioRender.com/gdrl3ym.

The translation of genetic knowledge to clinical care has the potential to enable precision medicine. Following the precision medicine EPPOS framework, we will discuss how genetic information can be used to aid diabetes diagnosis, risk prediction and classification, treatment selection, and monitoring (Fig. 1).^54^ Improved risk prediction for those at higher likelihood of developing diabetes can inform resource allocation and prevention strategies. Additionally, genetic data provide accurate molecular diagnosis for monogenic diabetes and aid diabetes classification, both required to optimise therapeutic regimens and improve quality of life. Lastly, appropriate interpretation of biomarker thresholds, informed by the impact of genetic variation, are essential for individual diagnosis, monitoring and appropriate treatment escalation, and to understand the population burden of diabetes.

### Molecular diagnosis facilitates tailored therapy

The management of monogenic diabetes is arguably one of the biggest successes of the application of genetics in diabetes care, highlighting the importance of making an accurate molecular diagnosis. This is a good example of how accurate personalised diagnosis affects treatment selection (Fig. 1).

NDM and MODY cases are often clinically misclassified as T1D due to their early age of onset. Identification of a pathogenic variant that explains disease occurrence in a patient not only provides an accurate diagnosis but is critical in implementing the appropriate therapy. For example, patients with NDM with mutations in *ABCC8* or *KNCJ11* can usually be effectively managed with oral sulphonylureas rather than insulin.^55^^,^^56^ Likewise, sulphonylureas are also standard of care for MODY patients with *HNF1A* or *HNF4A* pathogenic variants.^11^ In contrast, patients with MODY caused by *GCK* pathogenic variants usually have mild stable hyperglycaemia and low risk of microvascular complications and can be managed without pharmacologic treatment.^57^ The exception is pregnant women who should be treated with insulin if the foetus has not inherited the *GCK* variant to prevent the foetus from becoming large for gestational age.^58^

These advances in personalised care of people with monogenic diabetes have not, however, been extended to Africa. Successful cessation of oral hypoglycaemic therapy and transition from insulin to sulphonylurea therapy have been reported in individuals from Tunisia and Cameroon after identification of *GCK* and *HNF1A* pathogenic variants, respectively.^12^^,^^13^ However, the absence of accessible and appropriately tailored genetic testing in most of the continent makes it impossible to identify individuals with monogenic diabetes syndromes on any appreciable scale (Fig. 1).

To help prioritise expensive genetic testing to those individuals most likely to have MODY, MODY probability calculators have been developed. These use standard clinical features to predict which patients presenting with hyperglycaemia are most likely to have MODY and would therefore benefit from genetic testing. This approach is designed to limit expensive genetic testing whilst ensuring the benefits of targeted therapeutics could be realised. However, existing MODY calculators have been developed in European ancestry populations (https://www.diabetesgenes.org/) and have been reported to overestimate MODY in some multi-ethnic populations in the United States.^59^^,^^60^ Little data are available on their performance in African populations, further hampering their usage to prioritise patients for genetic testing in this region.

In summary, although the identification of a genetic mutation underlying monogenic diabetes is the exemplar of personalised medicine, the ability to implement this approach across Africa has been hampered by the lack of appropriate MODY prediction models, the expensive nature of genetic testing and lack of infrastructure and human resources to support these efforts.

### Use of genetic risk scores in risk prediction and diabetes classification

One of the major opportunities for clinical translation of GWAS is in developing genetic risk scores (GRS) for the prediction of diabetes or to aid disease classification. These scores provide a summary of heritable disease risk encoded in an individual's genome. However, the use of such GRS has come with an increased concern that such tools, built on research findings from predominantly European ancestry individuals, perform poorly among other populations, and would increase health disparities.^61^

#### T1D GRS for disease prediction

Several T1D GRS have been developed to determine an individual's risk of developing T1D. Risk prediction for the development of T1D can facilitate closer monitoring of at-risk individuals and reduce the likelihood of severe metabolic decompensation at the time of presentation, which is critical given the under-resourced health care systems in several parts of Africa.

While several T1D GRSs have been developed that differ in the number of HLA and non-HLA variants present, these are predominantly derived from European populations which may limit their applicability in genetically diverse African populations. A European ancestry derived T1D GRS (30 variants) had reduced predictive power in a self-identified African-American cohort (area under the curve [AUC]:0.752) compared to its application in self-identified White (AUC:0.860) and Asian Americans (AUC: 0.918).^62^ In contrast, a GRS consisting of 19 variants associated with T1D in European populations was able to discriminate between participants with T1D and controls (GRS: 0.189 vs. 0.154) in an Ethiopian population.^21^

The first African-ancestry specific GRS for T1D (AAGRS) was developed in 2019 from African-American data and consisted of seven single nucleotide polymorphisms-five in the HLA region and two non-HLA variants.^63^ Use of the AAGRS significantly improved the prediction of T1D risk compared to a European based GRS (GRS1) consisting of 30 variants (five HLA and 25 non-HLA) (AUC: 0.871 vs. 0.798).^64^ In addition, the AAGRS was able to differentiate between T1D and T2D (AUC: 0.787).^63^

A 67-variant GRS (GRS2), including risk alleles common in non-European populations and a broader spectrum of HLA variants, showed improved classification of T1D in self-identified non-Hispanic White (AUC: 0.864 vs. 0.851), Hispanic (AUC: 0.935 vs. 0.825) and Black participants (AUC: 0.851 vs. 0.807) from the SEARCH study in the United States compared to GRS1.^64^^,^^65^ The performance of each of GRS1, GRS2 and AAGRS was evaluated in the YODA (Cameroon and Uganda) and SEARCH datasets. GRS2 and AAGRS had significantly higher discriminative power than GRS1 in the Cameroonian (AUC: 0.876, 0.885 and 0.834, respectively), Ugandan (AUC: 0.890, 0.879 and 0.800, respectively) and African American populations (AUC: 0.839, 0.838 and 0.796, respectively).^66^ GRS2 had the greatest predictive power in the American White (AUC: 0.878) and Hispanic datasets (AUC: 0.868) whereas the AAGRS had the lowest power (AUC: 0.810 and 0.789, respectively) in these two groups.^66^ These findings suggest that multi-ancestry GRS may be more applicable in some African populations, but not necessarily others, and further evaluation in more diverse datasets is necessary.

#### T1D GRS for disease classification

T1D GRS have been useful in differentiating between individuals with clinical features that overlap between different diabetes types. Different versions of T1D GRS have been useful in differentiating individuals who have T1D from those with MODY or T2D.^64^^,^^67^ A successful application of the GRS2 was recently reported in Cameroonian, South African and Ugandan participants with youth onset, clinically diagnosed T1D. In this study, approximately 65% of clinically diagnosed T1D patients were found to be insulin deficient but autoantibody negative. These individuals had significantly lower genetic susceptibility to T1D measured by GRS2, compared to insulin deficient autoantibody positive patients (GRS: 9.66 vs. 11.76), suggesting a novel type of non-autoimmune insulin deficient diabetes subtype common in sub-Saharan Africa.^16^

#### T2D GRS for disease prediction

Unlike T1D where HLA alone captures a significant proportion of T1D risk, the risk for T2D is distributed among thousands of variants across the genome which overall capture the risk of T2D less well. Therefore, the clinical utility of T2D GRS for diabetes risk prediction is still debated.^68^ Nonetheless, the first evaluation of GRS for T2D in sub–Saharan Africa was done as part of a GRS study of 12 cardiometabolic traits comparing their performance in Africans to African Americans and European Americans.^69^ The predictive utility of GRS was tested in a sample of ∼25,000 individuals that included 5200 Africans, 9139 African Americans, and 9594 European Americans. For T2D, the GRS was associated with risk of T2D in all three groups but showed the lowest predictive utility in the Africans, though the measure of this difference is not provided.

A more recent study of GRS for T2D prediction in Africa tested the hypothesis that using African American, European, or multiethnic-derived GRS could improve prediction in continental Africans. The analysis showed that the discriminatory ability of the African American (AUC: 69.8%) and multiethnic GRS (AUC: 69.9%) was similar in a full model with clinical risk factors, but African American-derived GRS was more transferable in the countries represented in the continental African study (AADM) and was more predictive of T2D in the country combined analysis compared with the European and multiethnic-derived scores. The study showed that, given currently available data, African American-derived GRS enhanced prediction of T2D in some continental African populations.^70^

GDM risk scores have been developed but their clinical utility has not yet been validated for any population, and none have been based on data from continental Africa populations.^71^, ^72^, ^73^, ^74^

In summary, while there are ongoing efforts to develop T2D GRS and make these more relevant for populations around the world, more work is needed to increase their transferability and applicability to different African populations. In addition, the application of these scores for risk prediction, in combination with other clinical factors, is still an area of ongoing development.

### Impact of genetic variants on the accuracy of diabetes biomarkers for precision diabetes diagnosis and monitoring

Glycated haemoglobin (HbA1c) is the established biomarker for monitoring glycaemic control in individuals with diabetes and is increasingly used for diabetes diagnosis.^75^ HbA1c is an indirect measure of glucose, resulting from the non-enzymatic binding of glucose to haemoglobin inside the red blood cell.^75^ Therefore, HbA1c measurement can be affected by both glycaemic and non-glycaemic factors (for example factors that affect erythrocyte lifespan), including common genetic variants that associate with HbA1c without affecting glycaemia.^76^

Some of these non-glycaemic HbA1c-associated genetic variants are common across different African populations as they convey a selective advantage in malaria-endemic regions. These variants can affect the accuracy of HbA1c as a proxy measure of glucose and limit the applicability of universal HbA1c diagnostic and therapeutic thresholds. For example, the X-linked *G6PD* rs1050828 variant, (NM_000402.4:c.292G>A, p.Val98Met) which causes glucose-6-phosphate dehydrogenase (G6PD) deficiency, reduces the lifespan of the red blood cell and is associated with falsely low HbA1c levels. For any given glucose level, hemizygous men and homozygous women have 0.81% and 0.68% lower HbA1c levels, respectively which likely contributes to the delayed diabetes diagnosis, delayed treatment escalation and increased risk of diabetes complications in people with G6PD deficiency.^77^, ^78^, ^79^, ^80^, ^81^, ^82^, ^83^ Consistent with random X chromosome inactivation, heterozygous women have a more modest reduction in HbA1c (0.26%).^77^

G6PD deficiency is common in malaria-endemic regions of the world as it confers relative protection against severe malaria. Central sub-Saharan Africa has the highest incidence of G6PD deficiency worldwide, with the rs1050828 combined with the rs1050829 variant (NM_000402.4:c.466A>G, p.Asn156Asp) forming the A-haplotype that is common in the sub-continent.^84^, ^85^, ^86^, ^87^ Significant allelic heterogeneity exists, with other variants identified in several countries including Ethiopia, Senegal, The Gambia and Eritrea.^88^, ^89^, ^90^, ^91^, ^92^, ^93^, ^94^ Investigating the effect of these additional *G6PD* variants on HbA1c is needed to fully understand their potential impact on diabetes diagnosis and management.

Other common variants that protect against severe malaria in different African populations and that associate with HbA1c but not glucose levels include the α−3.7 thalassaemia deletion in a rural Ugandan population cohort, the sickle cell anaemia causal variant rs334 in *HBB,* and variant rs148228241 in an intron of *HBA1*.^45^^,^^76^^,^^95^ Notably, as many of these variants are associated with falsely low HbA1c measurement, delays in diabetes diagnosis or in treatment escalation can occur, leading to increased risk of complications.

In addition to the above-mentioned variants, common non-glycaemic genetic variants associated with lower HbA1c levels are associated with higher prevalence of retinopathy in European and African ancestry individuals, highlighting the clinical implications of the underestimation of glycaemia that can occur with these non-glycaemic HbA1c-associated variants.^96^

Overall, these findings highlight the importance of recognising the effects of genetic variants that alter the accuracy of HbA1c test as a proxy measure of glucose, so this information can be considered when diagnosing patients and making treatment decisions. However, additional work is required to understand the individual and public health impact of these variants, particularly in different populations in Africa where the prevalence of these genetic variants is hugely variable.

## Challenges in the implementation of diabetes-related precision medicine in Africa

Adequate study of the influence of genetics on diabetes in Africa would require the investment of significant financial, infrastructural and human resources. Multi-site studies would be necessary to truly capture the extent of the significant genetic diversity in the continent. In-country infrastructure and capacity development would be essential to ensure African scientists can collect, analyse and longitudinally store samples from their populations, and community engagement would be vital in ensuring participant communities fully understand and can benefit from research endeavours. As it stands, investment in research by African governments is low, with governments in sub-Saharan Africa spending 0.44% of their gross domestic product on research and development in 2007 and those in North Africa spending between 0.48 and 0.91% according to the most recent figures available from the World Bank.^97^

Clinical applications of novel or existing insights into the genetic aetiology of diabetes are, unfortunately, likely to remain inaccessible to much of the continent for some time. While testing for genetic variants has become cheaper, it is still prohibitively expensive for much of Africa, particularly when one considers that nearly a third of health care expenditure in sub-Saharan Africa is out-of-pocket.^98^ The potential benefits of personalised approaches to diabetes management must also be considered in the context of limited access to basic diabetes therapies. In 2021, the age-standardised treatment coverage for diabetes for adults in the World Health Organisation Africa region aged 30 years and older was 26%.^99^ Introduction of personalised approaches to diabetes management will only improve population outcomes if diabetes care as a whole is improved.

## Outstanding questions

Much remains unknown about the genetic aetiology of diabetes in Africa (Table 4). Further research initiatives across several different areas are necessary before the promise of diabetes precision medicine can begin to be fulfilled in the continent.

**Table 4:** Common genetic associations with diabetes in African and European populations and precision medicine implications.

Diabetes subtype	Common genetic associations in African populations	Common genetic associations in European population	Precision medicine implications
Neonatal diabetes	*EIF2AK3*^a^	*ABCC8*^b^; *KCNJ11*^b^	*EIF2AK3*: require insulin therapy; investigation for neurologic, skeletal and hepatic features *ABCC8*; *KCNJ11*: can be treated with sulphonylureas; investigation for neurologic features
Maturity onset diabetes of the young	Insufficient data available	*GCK*; *RFX6*; *HNF4A*; *HNF1A, HNF1B*^c^	*GCK*: managed with lifestyle modification *RFX6*: may be treated with non-insulin therapies *HNF4A*; *HNF1A*: highly sensitive to sulphonylureas
Type 1 diabetes	Greater genetic diversity with African specific haplotypes eg. HLA-DR9 Candidate gene studies with small sample sizes, GWAS rare and insufficiently powered	The greatest risk is conferred by the HLA DR3-DQ2 and DR4-DQ8 haplotypes Non-HLA include >75 risk loci, including susceptibility genes INS, CTLA4, IL2RA, IL10 and PTPN22	In black African populations the risk of misdiagnosis is high, ancestry-specific risk prediction tools are needed together with new diagnostic criteria and therapeutic strategies
Type 2 diabetes	Limited data; At genome wide significance, *TCF7L2* (in common with most other populations), *ZRANB3* and *AGMO* (novel locus in Africans); 32 replicated loci from previous studies.	Over 600 common genetic associations	Evidence base for GRS for T2D prediction in Africa is poor and its potential clinical utility is currently unknown

a In populations with high prevalence of parental consanguinity.

b In populations with a low prevalence of parental consanguinity.

c Prevalence based on an unselected population based cohort.^100^

Given the huge genetic diversity across the African continent, the backbone of future research will be more complete characterisation of the genetic architecture in different African populations through the generation of large-scale sequencing data and complex phenotyping from large cohorts of unselected individuals from diverse populations. These datasets will enable rare variant interpretation in monogenic diabetes, and potentially help identify additional variants that are responsive to tailored therapy. Larger-scale GWAS in patients with type 1 and type 2 diabetes from across the continent will help elucidate relevant pathophysiological pathways which may be unique to certain populations in Africa, given the continent's unique population history, diverse geographical features and highly variable environmental exposures.^16^

To aid physiologic and pathophysiologic characterisation of the different types of diabetes present across different populations in the African continent, deep phenotyping of patients and unaffected individuals will be required. Such phenotyping should include detailed physiologic and imaging studies, as well as further OMIC characterisation in both affected and unaffected individuals (e.g. transcriptomic, proteomic and metabolomic) which will enable more in depth understanding of causal mechanisms and pathways and may identify novel therapeutic targets.

Furthermore, detailed characterisation of the number and prevalence of genetic variants that affect the accuracy of the HbA1c measurement in populations across Africa, is crucial to understand the impact they are having on diagnosis and treatment. Adequate genotype-informed interpretation of HbA1c diagnostic and therapeutic thresholds may be required, or use of alternative biomarkers may be needed, to prevent increased risk of diabetes complications.

Finally, it is critical to expand initiatives to develop genomic medicine expertise in Africa, such as those of The African Genomic Medicine Training Initiative,^101^ to ensure effective translation from genetic discovery to bedside.

**Search strategy**

Articles for this Review were identified by PubMed searches and included articles from database inception to September 2025, using terms “Diabetes mellitus/genetics” [Mesh] AND “Africa”. Additional search terms included “monogenic diabetes”, “type 1 diabetes”, “HLA”, “type 2 diabetes”, “gestational diabetes”, “HbA1c”, “glucose-6-phosphate deficiency” each combined with Boolean term AND “Africa”. Articles that were not in English, where the full text could not be accessed, that were not human studies based on titles and abstracts were excluded. Articles were evaluated to confirm that they contained original human genetic research and were studies done on the African continent, those where this was not the case were excluded. Additional references were obtained from references in relevant articles. Additional references citing other reviews and diabetes studies from across the world were also included as background and context.

## Contributors

All authors performed literature searches. IB and CP drafted Fig. 1, CP drafted Tables 1 and 2, AA drafted Table 3, AW drafted Table 4. All the authors drafted different sections of the manuscript. All the authors edited, read and approved the final version of the manuscript, including Tables and Figure.

## Declaration of interests

AW declares support from Novo Nordisk to attend a medical education event. AW is a clinical co-chair for the Research Affairs Core Committee of the Endocrine Society and a member of the Diabetes Guidelines Committee for the Society for Endocrinology, Metabolism and Diabetes of South Africa. AA is funded by National Institutes of Health (NIH) through the Center for Research on Genomics and Global Health (CRGGH). IB declares support from MRC (MR/W014416/1) and infrastructure support from the National Institute for Health and Care Research Exeter Biomedical Research Centre. The views expressed are those of the authors and not necessarily those of the NIHR or the Department of Health and Social Care. IB declares support from Diabetes UK for travel and conference registration expenses for delivery of Dorothy Hodgkin Prize Lecture 2025, and from the European Association for the Study of Diabetes for conference registration and travel expenses as an invited symposium speaker at the 2025 meeting. IB was Co-chair for Wellcome Career Development Interview Committee until September 2025, and is member of Diabetes UK research committee, and is faculty of Diabetes UK IDia programme.
